# A novel 2B4 receptor leads to worse pregnancy outcomes by facilitating TNF-α and IFN-γ production in dNK cells during *Toxoplasma gondii* infection

**DOI:** 10.1186/s13071-022-05455-9

**Published:** 2022-09-24

**Authors:** Xiaoyan Xu, Guangmei Zheng, Yushan Ren, Xiaohua He, Biwen Peng, Xuemei Hu, Wanhong Liu

**Affiliations:** 1 Hubei Province Key Laboratory of Allergy and Immunology, Department of Immunology, School of Basic Medical Sciences, Wuhan University, No. 185, Donghu Road, Wuchang District, Wuhan, 430071 China; 2 Department of Immunology, Binzhou Medical University, No. 346, Guanhai Road, Laishan district, Yantai, Shandong China

**Keywords:** 2B4, Pregnancy failure, TNF-α, IFN-γ, *Toxoplasma gondii* infection, Decidual NK cells, Pathway

## Abstract

**Background:**

Infections are a major threat to human reproductive health because they can induce pregnancy failure, including recurrent abortion, stillbirth, and preterm birth. *Toxoplasma gondii* (*T. gondii*) infection can result in adverse pregnancy outcomes by affecting certain immune molecules and cytokines. However, the detailed mechanisms behind *T. gondii-*induced pregnancy failure are poorly understood.

**Methods:**

*Toxoplasma gondii*-infected wild-type (WT) pregnant mice and 2B4 knockout (2B4^−/−^) pregnant mice were established for in vivo study. Human decidual natural killer (dNK) cells were cultured for in vitro study. Abnormal pregnancy outcomes were observed, and the expression of 2B4, functional molecules (CD69, CD107a, tumor necrosis factor alpha [TNF-α], interferon gamma [IFN-γ]), and signaling molecules (SHP-2, Fyn, p-ERK, p-P38) in dNK cells were detected by flow cytometry, Western blot, reverse transcriptase polymerase chain reaction (RT-PCR), and/or immunofluorescence. The direct interactions (2B4 interacts with SHP-2 and Fyn; SHP-2 interacts with p-P38 and 2B4; Fyn interacts with p-ERK and 2B4) were verified by co-immunoprecipitation (co-IP) in NK-92 cells.

**Results:**

Here, results showed that 2B4 was significantly downregulated after *T. gondii* infection. Subsequently, infected 2B4^−/−^ pregnant mice displayed worse pregnancy outcomes compared with infected WT pregnant mice. Also, increased TNF-α and IFN-γ expression and elevated dNK cell cytotoxicity were found in 2B4^−/−^ pregnant mice during *T. gondii* infection. In contrast, reduced TNF-α and IFN-γ expression and decreased human dNK cell activity were found following 2B4 activation during *T. gondii* infection. Interestingly, results showed that 2B4 binds to adaptor SHP-2 or Fyn, which then triggers different signaling pathways to regulate TNF-α and IFN-γ expression in dNK cells during *T. gondii* infection. Further, SHP-2 binds 2B4 and p-P38 directly after 2B4 activation, which generates an inhibitory signal for TNF-α and IFN-γ in NK-92 cells. In addition, Fyn can bind to 2B4 and p-ERK after activation of 2B4, thereby inhibiting TNF-α and IFN-γ expression in NK-92 cells following *T. gondii* infection.

**Conclusions:**

These data suggest that 2B4 may be a novel danger-signaling molecule that is implicated in pregnancy failure during *T. gondii* infection. Unraveling the mechanism by which 2B4 regulates dNK cell activity will provide novel insights to aid our understanding of *T. gondii*-induced adverse pregnancy outcomes.

**Graphical Abstract:**

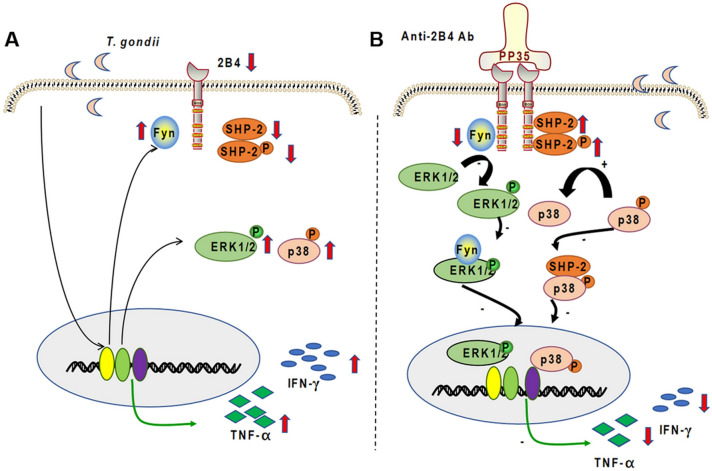

**Supplementary Information:**

The online version contains supplementary material available at 10.1186/s13071-022-05455-9.

## Background

During pregnancy, and particularly during early pregnancy, various abnormalities may lead to adverse pregnancy outcomes, including but not limited to recurrent abortion, preterm birth, stillbirth, fetal growth restriction, and preeclampsia [1–4]. Due to complex environmental factors, infections are a major threat to pregnancy outcomes, and approximately 10–30% of all stillbirths have an infectious etiology [5–8]. *Toxoplasma gondii* (*T. gondii*), a global zoonotic parasite, is the only parasite of the TORCH (*T. gondii*, other, rubella virus, cytomegalovirus, herpes simplex virus) family that poses a threat to public health [9]. Congenital *T. gondii* infection is believed to be acquired through the placenta during pregnancy and can result in congenital toxoplasmosis in newborns [10]. However, preconception infection can occasionally lead to implications for the fetus in immunodeficient women [11]. In addition, during pregnancy, protection against *T. gondii* can be breached after reinfection with parasites belonging to different genotypes, particularly when non-clonal strains are involved in this process, and in this case the reinfection promotes vertical transmission of *T. gondii* [12]. Normal pregnancy is an intricately orchestrated process that requires maintenance of immune tolerance at the fetal–maternal interface. The mechanisms behind pregnancy loss in *T. gondii* infection may be mediated by dysfunction of the immune microenvironment [13, 14]. But the detailed mechanism of how *T. gondii* infection induces abnormal pregnancy outcomes remains unclear. Therefore, there is an urgent need to explore the specific mechanism(s) or common mechanism(s) responsible for inducing pregnancy failure, as these may provide novel insights to help reduce or prevent the incidence of adverse pregnancy outcomes after *T. gondii* infection.

Various immune cells and molecules in the immune microenvironment establish specific maternal tolerance toward the semi-allogeneic fetus during early pregnancy [15]. Among various immune cells, decidual natural killer (dNK) cells are the most distinguishable lymphocytes during the first trimester of pregnancy, constituting > 70% of all leukocytes in the human decidua [16]. In our previous study, we showed that, following *T. gondii* infection, disturbance of the dNK cell function induced abnormal pregnancy outcomes during early pregnancy [17, 18]. However, the detailed molecular mechanism involved in regulating dNK cell function during the occurrence of adverse pregnancy outcomes caused by *T. gondii* infection needs to be further explored. 2B4 (CD244), a member of the signaling lymphocyte activation molecule (SLAM) family receptors, was first identified on mouse NK cells and a subset of T cells that mediate non-major histocompatibility complex (MHC)-restricted cytotoxicity [19]. Previous studies have suggested that 2B4 plays multiple complex immune roles in tumor immune escape [20, 21]. High 2B4 expression or loss can promote immune cell depletion, leading to the conversion of acute Epstein–Barr virus (EBV) infection to a chronic infection [22–24]. However, there is little reported about the relationship between 2B4 and abnormal pregnancy outcomes. Taken together, these findings have prompted us to investigate the role of 2B4 in abnormal pregnancy outcomes induced by *T. gondii* infection.

Herein, we generated 2B4^−/−^ mice and found a novel receptor, 2B4, which acts as an important immune molecule in abnormal pregnancy outcomes following *T. gondii* infection. The specific mechanisms by which 2B4 induces adverse pregnancy after *T. gondii* infection require further investigation. This study will provide new insights into our current understanding of *T. gondii*-induced adverse pregnancy outcomes.

## Methods

### Generation of mouse model

All 2B4^−/−^ mice (C57BL/6 background) were constructed by Nanjing Aipu Xilong Medical Technology Co., Ltd. (Nanjing, China), and C57BL/6 wild-type (WT) mice were purchased from Pengyue Laboratory Animal Technology Co., Ltd. (Jinan, China). All mice were kept in the specific pathogen-free animal house of Binzhou Medical University. WT 8–10-week-old males were matched with 6–8-week-old WT females. Male 2B4^−/−^ mice were also matched with female 2B4^−/−^ mice at a ratio of 1:2 overnight, and female mice with vaginal plugs at gestational day (gd) 0 were segregated and randomized into uninfected mice (32 mice), infected WT mice (39 mice), and infected 2B4^−/−^mice (21 mice). Infected WT mice and infected 2B4^−/−^ mice were intraperitoneally injected at gd 6 with 400 tachyzoites (RH strain) resuspended in 200 μl of phosphate-buffered saline (PBS). At gd 11, the mice were euthanized by an isoflurane–ketamine/xylazine mixture to detect the expression of 2B4, CD69, tumor necrosis factor alpha (TNF-α), and interferon gamma (IFN-γ) in dNK cells using flow cytometry. All methods of euthanasia conformed to the current 2013 American Veterinary Medical Association guidelines [25]. All animal experiments and handling procedures were approved by the ethics committee of Binzhou Medical University and Wuhan University and were performed in accordance with their guidelines (Protocol 2018-10-021).

### *Toxoplasma gondii *tachyzoites (RH strain) and GFP-*T. gondii* preparation

Human foreskin fibroblast (HFF) cells were obtained from the Anhui Medical University (Anhui, China) and used to amplify *T. gondii.* HFF cells were cultured in 90% Dulbecco’s modified Eagle medium (DMEM) with high sugar content (HyClone, USA) containing 10% fetal bovine serum (FBS; Gibco, USA) and infected with *T. gondii.* After infection for 48 h, the supernatants were collected and centrifuged at 800 rpm for 5 min at room temperature to remove the HFF cells. Purified tachyzoites from supernatants were then acquired by centrifugation at 4000 rpm for 7 min.

### dNK cell separation from mice and humans

dNK cells were separated from tissue taken from patients undergoing voluntary abortion in the Yantai Maternal and Child Health Hospital and Department of Obstetrics and Gynecology of Yantai Affiliated Hospital of Binzhou Medical University. All voluntary abortions occurred during the first trimester, between 6 and 12 weeks of gestation. The inclusion criteria for samples were similar to those we described previously [17]. Sample collection procedures for this study were approved by the ethics committee of Binzhou Medical University and Wuhan University, and informed consent was obtained from all patients. This study was conducted according to the principles expressed in the Declaration of Helsinki. Mice uteri and placentas or human decidual tissues were carefully dissected from pregnant mice at gd 11, and deciduae from patients were washed several times in cold PBS. Tissues were then cut into small pieces and digested with 0.1% collagenase type IV (Sigma, Germany) and 25 IU/ml DNase-I (Sigma, Germany) for 1 h at 37 °C with shaking. Sterile nets (48 µm) were used to obtain single-cell suspensions. Mononuclear cells were collected from the white film layer after Ficoll density gradient centrifugation in lymphocyte separation medium (TBD Science, China). Mouse and human peripheral blood mononuclear cells (PBMCs) were used for flow cytometry detection. We also purified dNK cells using a human CD3-positive selection kit and a CD56-positive selection kit (Stem Cell Technologies, Canada) according to the manufacturer’s instructions, with > 85% purity ensured for Western blotting. To clarify the role of 2B4 and the specific binding molecules involved in dNK cells, cells were divided into three groups (approximately 1 × 10^7^ cells per group): uninfected group, infected group (at a 1:5 ratio of *T. gondii*: cells), and anti-2B4 antibody-treated infected group (5 μg/ml; Invitrogen, USA). To explore the role of SHP-2 or Fyn, cells were divided into the following groups: infected group, anti-2B4 antibody-treated infected group, anti-2B4 antibody- and SHP-2 inhibitor-treated infected group, and anti-2B4 antibody- and Fyn inhibitor-treated infected group. All samples were cultured in RPMI [Roswell Park Memorial Institute] medium supplemented with 10% FBS (Gibco, USA) and 100 IU/ml penicillin/streptomycin (Sigma, Germany) for 20 h at 37 °C in a humidified 5% CO_2_ incubator.

### NK-92 and K562 cell cultures

NK-92 cells were cultured in RPMI 1640 medium (HyClone, USA) with 10% FBS for co-immunoprecipitation (co-IP) assays. NK-92 cells were divided into three groups: uninfected group, infected group, and anti-2B4 antibody-treated infected group. K562 cells were also cultured in RPMI 1640 medium (HyClone, USA) with 10% FBS and used as a target cell for killing experiments.

### Flow cytometry assay

For experiments measuring 2B4, TNF-α, IFN-γ, and CD69 in mouse dNK cells, the following fluorochrome-conjugated monoclonal antibodies (mAbs) were used: APC-CY7-CD45 (BioLegend, USA), PERCP-CY5.5-CD3e (Invitrogen, USA), APC-CD122 (Invitrogen, USA), FITC-CD49b (BioLegend, USA), BV421-2B4 (BD Pharmingen, USA), PE-TNF-α (Invitrogen, USA), PE-IFN-γ (Invitrogen, USA), and PE-CD69 (BioLegend, USA). In vitro, human-specific mAbs were used: APC-CY7-CD45 (BioLegend, USA), FITC-CD3 (BD Pharmingen, USA), PERCPCY5.5-CD56 (Invitrogen, USA), APC-2B4 (Invitrogen, USA), BV421-CD48 (BD Pharmingen, USA), BV421-CD107a (BD Pharmingen, USA), PE-TNF-α (Invitrogen, USA), PE-IFN-γ (BD Pharmingen, USA), and PE-IgG (BD Pharmingen, USA). Isolated murine dNK cells from the uninfected group, infected WT group, and infected 2B4^−/−^ group were stained for CD45, CD3e, CD122, CD49b, 2B4, and CD69 to detect 2B4 and CD69 expression on dNK cells. Human decidual PBMCs from three groups (uninfected group, infected group, and anti-2B4 antibody-treated infected group) were collected and stained for CD45, CD3, CD56, 2B4, and CD48 expression at 4°C in the dark for 30 min and then washed with PBS for flow cytometry. Isolated dNK cells from mice and humans were incubated with brefeldin A cocktail (MCE, China) at 37 °C for 6 h. The stimulated dNK cells were then stained with surface antibodies, ruptured by Foxp3/Transcription Fix/Perm buffer (Invitrogen, USA) overnight, and incubated with TNF-α and IFN-γ antibody in succession for 3 h at 4 °C. Data from flow cytometry were analyzed by FlowJo 10.7 software (Becton Dickinson, USA). In addition, 5 × 10^5^ PBMCs were stained with BV421-conjugated anti-CD107a and incubated with K562 cells at an effector/target (E:T) ratio of 5:1 in the infected group and anti-2B4 antibody-treated infected group.

### Reverse transcriptase polymerase chain reaction (RT-PCR) for CD244, SHP-2

RNA was isolated from uninfected and infected dNK cells using TRIzol and chloroform and then precipitated using isopropanol. Generation of cDNA was accomplished using a Thermo Fisher High-Capacity cDNA Reverse Transcription Kit following the manufacturer’s instructions, and RT-PCR was catalyzed using an RT-PCR synthesis kit (Qiagen, Germany). The following primers were used: h2B4-A: F: 5′-CCTCTACTGCCTGGAGGTCACCAG-3′, R: 5′-CCACTTGGCATCTCCCTCTGTCC-3′; h2B4-B: F: 5′-GCCTTCCAATACTTCC-3′, R: 5’-TGGAAGCAGAGATTC-3′; SHP-2: F: 5′-AAAGGGGAGAGCAATGACGG-3′, R: 5′-GGGGCTGCTTGAGTTGTAGT-3′. Measurements were normalized to actin: F: 5′-GCACCGTCAAGGCTGAGAAC-3′, R: 5′-TGGTGAAGACGCCAGTGGA-3′.

### Immunofluorescence analysis

Purified human CD3^−^CD56^+^ dNK cells from uninfected and infected groups were collected after 20 h infection. All cells were washed and fixed for 30 min with 4% paraformaldehyde (PFA). Cells were incubated with APC-conjugated 2B4 antibody for 30 min at 4 °C and then washed. Following washing, the slides were mounted with reagent containing 4′,6-diamidino-2-phenylindole (DAPI) stain. Cells were placed in the confocal chamber, and images were captured by confocal fluorescence microscopy (Zeiss LSM 880).

### Inhibition of pathways assay

To understand whether the SHP-2/p-P38 and Fyn/p-ERK pathways are involved in 2B4-induced changes in TNF-α and IFN-γ following *T. gondii* infection, we used an SHP-2 inhibitor (MCE, China) and a Fyn inhibitor (Selleck, China), respectively. Firstly, untreated cells from the infected group were compared with infected cells treated with the SHP-2 inhibitor or infected cells treated with the Fyn inhibitor group to explore the role of SHP-2 and Fyn in downstream signaling affecting TNF-α and IFN-γ expression, respectively. In addition, to further confirm this, dNK cells were divided into the following three groups: infected group, anti-2B4 antibody-treated infected group, and anti-2B4 antibody- and SHP-2 inhibitor-treated infected group or anti-2B4 antibody- and Fyn inhibitor-treated infected group. In addition, to validate the effect of p-ERK and p-P38 on TNF-α and IFN-γ, dNK cells were divided into three groups: infected group, p-ERK inhibitor-treated infected group (MCE, China), or p-P38 inhibitor-treated infected group (MCE, China).

### LDH release assay

Purified dNK cells from infected dNK cells and anti-2B4 antibody-treated infected dNK cells were incubated with K562 cells at an effector/target (E:T) ratio of 5:1 for 6 h. Lactate dehydrogenase (LDH) production was measured in the supernatants using the LDH cytotoxicity assay kit (Beyotime, China). The data are presented as the percentage of the total LDH present relative to lysates of unstimulated cells.

### Co-immunoprecipitation assay

To investigate the interaction between 2B4 and SHP-2 or Fyn in the infected group with or without anti-2B4 antibody treatment, we used an anti-2B4 (Cell Signaling Technology, USA), anti-SHP-2 (Abcam, England), or anti-Fyn antibody (Abcam, England) for immunoprecipitation (IP). Cells from the uninfected group, infected group, and anti-2B4 antibody-treated infected group were collected and lysed in IP lysis buffer (Beyotime Technology, China) supplemented with protease inhibitors (Beyotime Technology, China) on ice for 45 min and centrifuged at 12,000 rpm at 4 °C for 15 min. Next, the supernatants were collected and incubated with 1 µg of the appropriate antibody at 4 °C overnight and precipitated with protein A/G-agarose beads (Beyotime Technology, China) for another 7 h at 4 °C. The beads were then washed with lysis buffer three times by centrifugation at 12,000 rpm at 4 °C. The precipitated proteins were denatured in 5 × loading buffer and analyzed by Western blotting.

### Western blot analysis

Purified dNK cells from all groups were incubated for 20 h before harvesting. Equal amounts of protein from total-cell lysates were separated by 10% sodium dodecyl surface-polyacrylamide gel electrophoresis (SDS-PAGE; Beyotime, China) and transferred onto polyvinylidene fluoride (PVDF) membranes (Millipore, USA). The membranes were then blocked at room temperature for 3 h in 7% (w/v) nonfat dry milk dissolved in tris-buffered saline/Tween^®^ 20 (TBS-T) buffer. Membranes were incubated with primary antibodies for 2B4 (Bioss, China), p-2B4 (Bioss, China), SHP-2 (Proteintech, China), Fyn (Proteintech, China), p-ERK (Abcam, England), p-P38 (Abcam, England), IFN-γ (Bioss, China), TNF-α (Bioss, China), horseradish peroxidase (HRP)-goat-anti-rabbit immunoglobulin G (IgG) (Proteintech, China), HRP-goat-anti-mouse IgG (Proteintech, China), and glyceraldehyde 3-phosphate dehydrogenase (GAPDH; Proteintech, China) overnight at 4 °C with gentle rocking. Membranes were washed with TBS-T five times and then incubated with the appropriate secondary antibody for 3 h at room temperature. Immune complexes were then visualized with an enhanced chemiluminescence (ECL) detection kit (F. Hoffmann-La Roche, Ltd., Switzerland). Protein expression levels were quantified in ImageJ software (Rawak Software, Inc., Germany).

### Statistical analyses

Data are presented as the mean ± standard deviation (SD). Statistical analyses were performed using the GraphPad Prism 7 Statistics software package. Unpaired and paired *t*-tests were used to identify differences, with a *P* value < 0.05 considered to be significant.

## Results

### 2B4^−/−^ pregnant mice show worse pregnancy outcomes following ***T. gondii*** infection

**Fig. 1:**
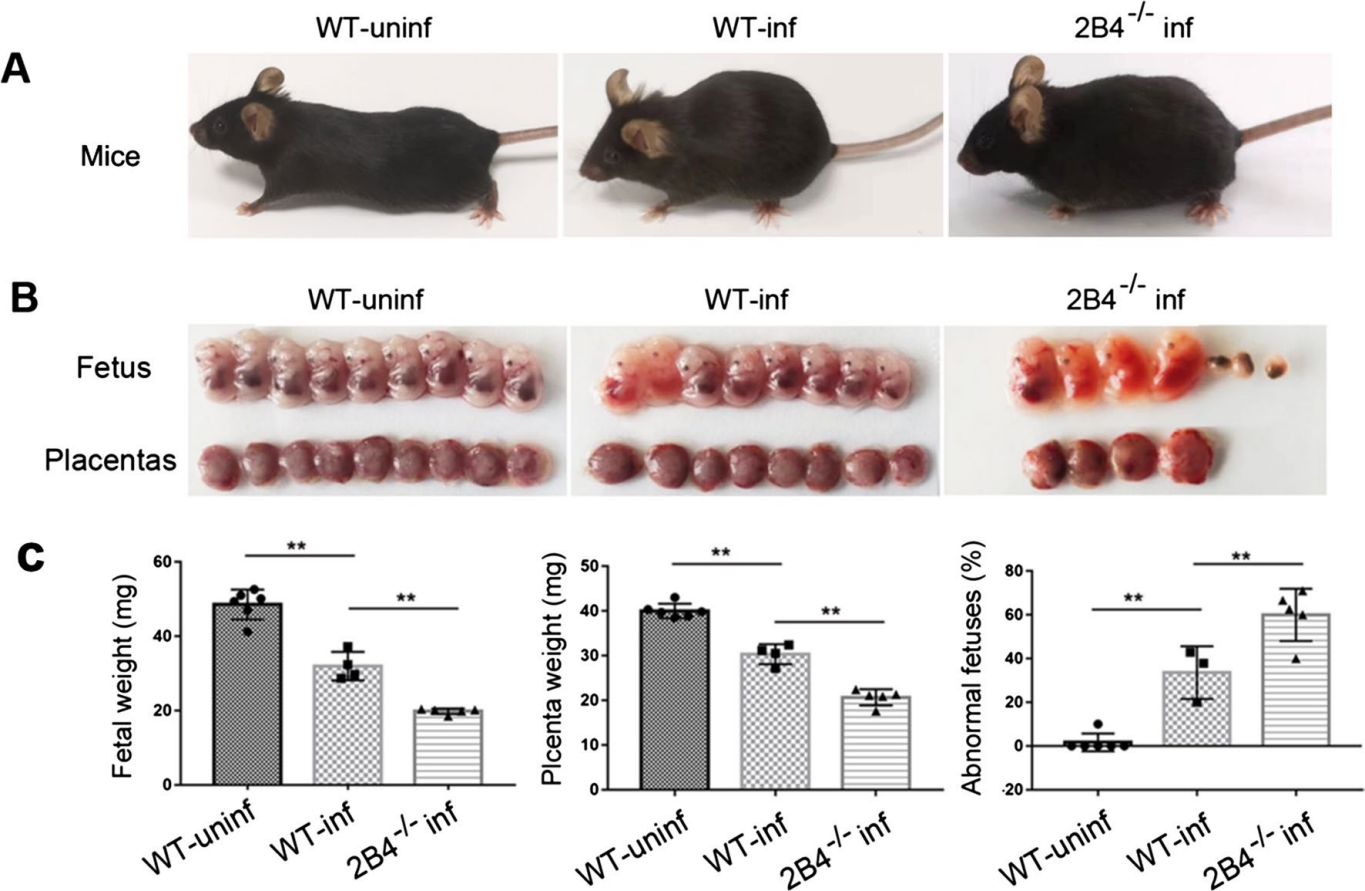
Pregnant infected 2B4^−/−^ mice exhibit worse pregnancy outcomes than infected WT mice. **A** Uninfected pregnant mice were in good condition; *T. gondii*-infected pregnant mice exhibited ruffled fur and depressive symptoms compared with uninfected pregnant mice, while *T. gondii*-infected 2B4^−/−^ pregnant mice had sluggish responses along with trembling compared with *T. gondii*-infected pregnant mice. **B** The pregnancy outcomes, including fetus and placenta, were observed by the naked eye in uninfected WT mice, infected WT mice, and infected 2B4^**−/−**^ mice (infected WT mice compared with uninfected mice; infected 2B4^−/−^ mice compared with infected WT mice). **C** The weight per fetus and placenta and the proportion of abnormal pregnancies were calculated in uninfected WT mice (*n* = 6), infected WT mice (*n* = 4), and infected 2B4^−/−^ mice (*n* = 5) (data are presented as the mean ± SD, ** *P* < 0.01, by unpaired *t*-test)

2B4^−/−^ mice were generated and identified by PCR using specific primers (Additional file 1: Fig. S1). Compared with infected WT mice, more serious abnormal pregnancy outcomes were found in infected 2B4^−/−^ mice, with ruffled fur and depressive symptoms (Fig. 1a). We observed worse pregnancy outcomes in infected 2B4^−/−^ mice, with a higher ratio of fetal death or resorption and more serious necrosis of placentas relative to infected WT mice (Fig. 1b). The fetal and placenta weights decreased and the percentage of abnormal fetuses significantly increased in infected 2B4^−/−^ mice compared with infected WT mice (Fig. 1c).

### 2B4 is impaired in mice dNK cells during *T. gondii* infection

**Fig. 2:**
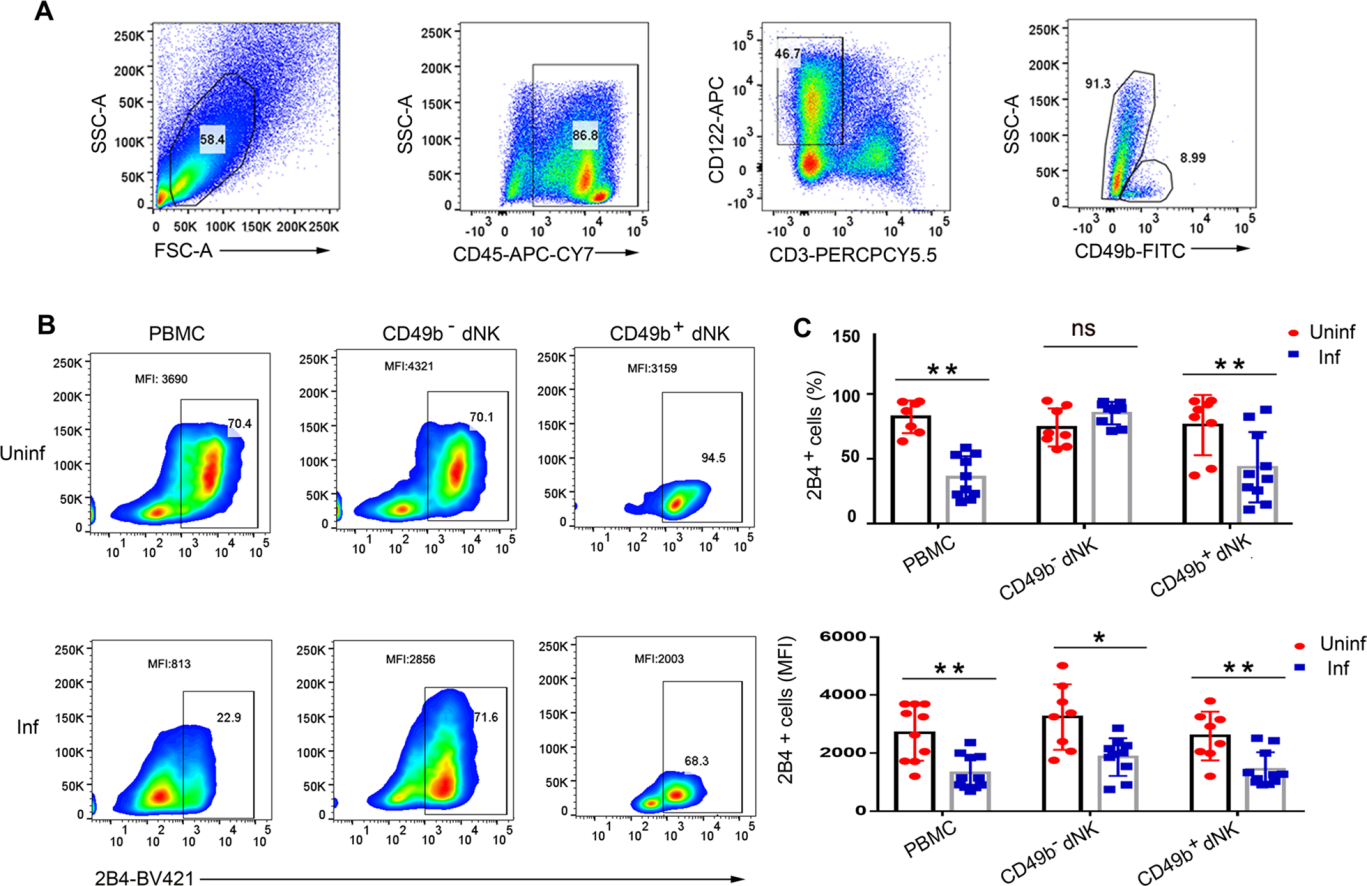
Impaired 2B4 expression on mouse dNK cells after *T. gondii* infection. **A** CD45^+^CD3^−^CD122^+^ represents dNK cells, and CD49b was characterized into the subset marker using flow cytometry. **B**, **C** The expression of 2B4 on the surface of immune cells, CD49b^−^ dNK, and CD49b^+^ dNK in uninfected WT mice (*n* > 8) and infected WT mice (*n* > 9) was detected by flow cytometry (data are presented as the mean ± SD; ** *P* < 0.01, *P < 0.05; by unpaired *t*-test)

CD45-positive cells were considered as immune cells, and CD3^−^CD122^+^ cells in immune cells were considered as dNK cells in mice (Fig. 2a). *Toxoplasma gondii* infection downregulated 2B4 expression in decidual immune cells, CD45^+^CD3^−^CD122^+^CD49b^−^ dNK cells, and CD45^+^CD3^−^CD122^+^CD49b^+^ dNK cells (Fig. 2b, c). In addition, dNK cells were collected, purified, and infected with green fluorescent protein-expressing (GFP)-*T. gondii* RH strain. Compared with GFP^−^ dNK cells in the infected group, a reduction of 2B4 was found in GFP^+^ dNK cells in the infected group (Additional file 1: Fig. S2).

### Upregulation of TNF-α and IFN-γ in infected 2B4^−/−^ pregnant mice triggers cytotoxic activity of dNK cells

**Fig. 3:**
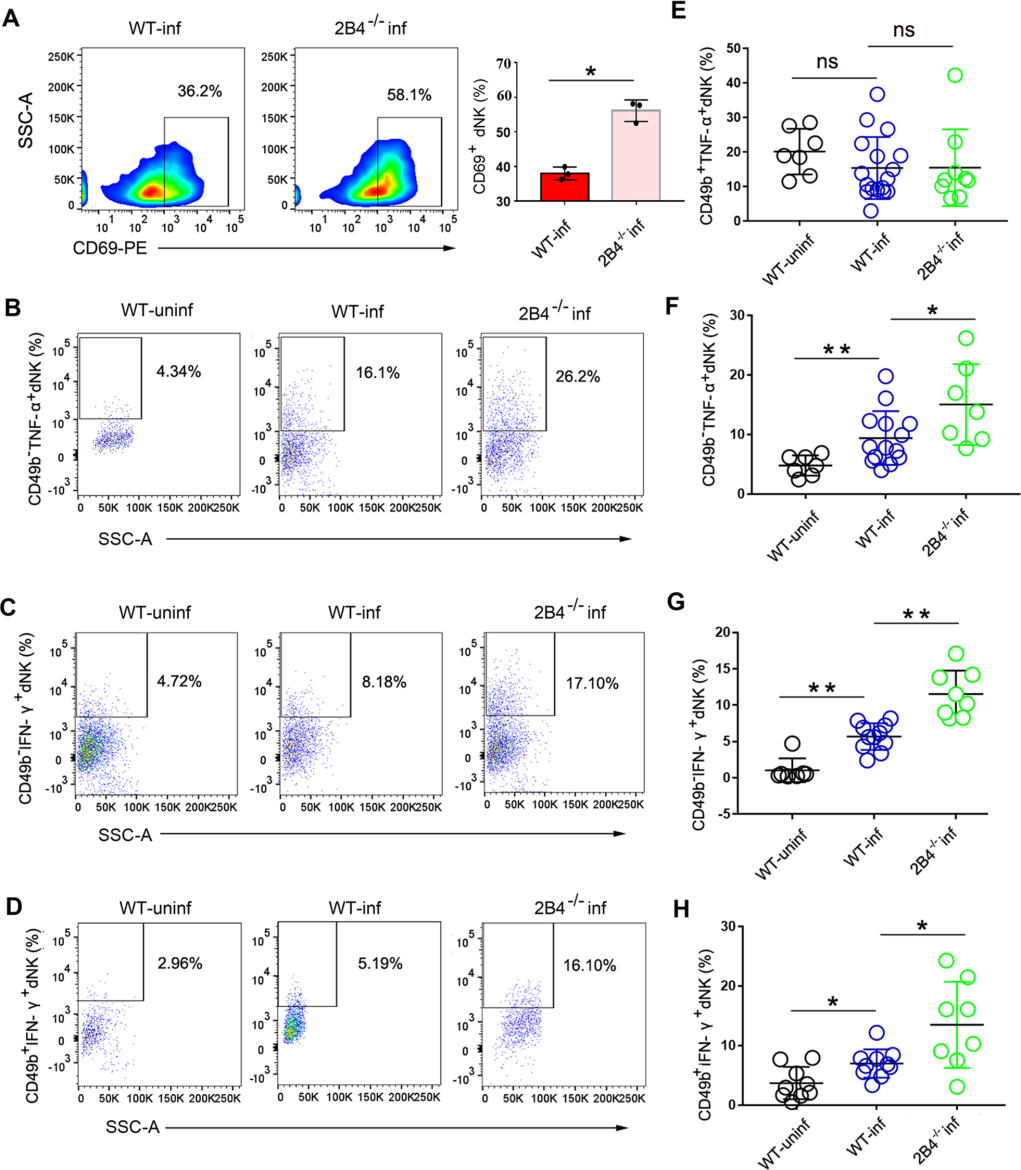
Dysfunction in dNK cells in infected 2B4^−/−^ pregnant mice leads to increased TNF-α and IFN-γ expression. **A** CD69 expression in infected WT mice and infected 2B4^−/−^ mice was detected by flow cytometry (data are presented as the mean ± SD, *n* = 3, *P < 0.05, by unpaired *t*-test). **B**, **F** Percentage of TNF-α^+^CD45^+^CD3^−^CD122^+^CD49b^−^ dNK cells in uninfected mice (*n* = 7), infected WT mice (*n* = 14), and infected 2B4^−/−^ mice (*n* = 7) (data are presented as the mean ± SD, **P < 0.01, *P < 0.05, by unpaired *t*-test). **E** Percentage of TNF-α^+^CD45^+^CD3^−^CD122^+^CD49b^+^ dNK cells in uninfected mice (*n* = 7), infected WT mice (*n* = 17), and infected 2B4^−/−^ mice (*n* = 9) (data are presented as the mean ± SD; *P* > 0.05 [WT-uninfected vs. WT-infected], *P* > 0.05 [WT-infected vs. 2B4^−/−^infected], by the unpaired *t*-test). **C**, **G** IFN-γ expression in the CD45^+^CD3^−^CD122^+^CD49b^−^dNK subset from uninfected mice (*n* = 7), infected WT mice (*n* = 10), and infected 2B4^−/−^ mice (*n* = 8) (data are presented as the mean ± SD; *P < 0.05 [WT-uninfected vs. WT-infected], *P < 0.05 [WT-infected vs. 2B4^−/−−^infected], by unpaired *t*-test). **D**, **H** IFN-γ in CD45^+^CD3^−^CD122^+^CD49b^+^ dNK cell subset from uninfected WT mice (*n* = 9), infected WT mice (*n* = 10), and infected 2B4^−/−^ mice (*n* = 8). Data are presented as the mean ± SD; *P < 0.05 (WT-uninfected vs. WT-infected), *P < 0.05 (WT-infected vs. 2B4^−/−^infected), by unpaired *t*-test

CD69 expression is readily upregulated upon activation in most leukocytes, which underlies its widespread use as a marker of activated lymphocytes, especially in NK cells [26]. We found that CD69 expression increased in dNK cells in infected 2B4^−/−^ mice compared with dNK cells from infected WT mice (Fig. 3a). Further, we observed increased TNF-α expression in the CD45^+^CD3^−^CD122^+^CD49b^−^ dNK subset from infected 2B4^−/−^ mice compared with that from infected WT mice (Fig. 3b, f). However, there were no differences in TNF-α expression in the CD45^+^CD3^−^CD122^+^CD49b^+^ dNK cells from infected WT mice and from infected 2B4^−/−^ mice (Fig. 3e). In addition, IFN-γ expression in the CD45^+^CD3^−^CD122^+^CD49b^−^ dNK subset (Fig. 3c, g) and CD45^+^CD3^−^CD122^+^CD49b^+^ dNK subset (Fig. 3d, h) was significantly increased in 2B4^−/−^ mice following *T. gondii* infection compared with infected WT mice.

### *Toxoplasma gondii* infection decreases expression of 2B4 and p-2B4, resulting in activation of cytotoxicity of human dNK cells

**Fig. 4:**
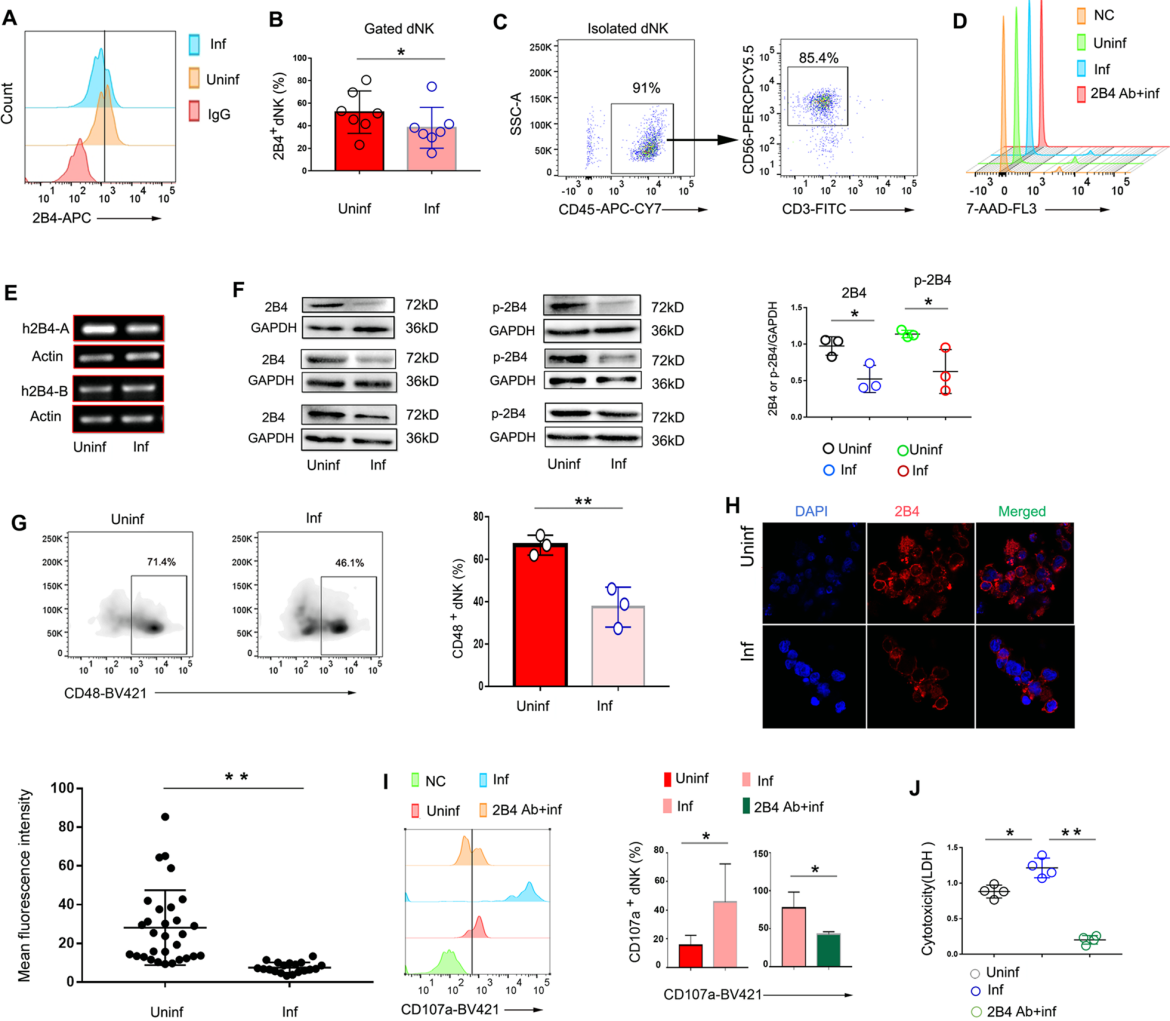
Downregulation of 2B4 and p-2B4 is associated with human dNK cell dysfunction during *T. gondii* infection. **A**, **B** PBMCs were isolated from healthy abortions from early pregnancy and stimulated with *T. gondii* for 20 h. 2B4 expression on the surface of dNK cells in the uninfected and infected groups (data are presented as the mean ± SD, *n* = 7, *P < 0.05, by the paired *t*-test). **C** Purified dNK cells were identified by flow cytometry. **D** 7-AAD was used to excluded the influences of dead cells. **E** *h2B4-A* and *h2B4-B* mRNA levels in uninfected and infected dNK cells. **F** Western blot analysis of 2B4 and p-2B4 in uninfected and infected dNK cells (data are presented as the mean ± SD, *n* = 3, *P < 0.05, by the paired *t*-test). **G** Expression of 2B4 ligand CD48 on dNK cells (data are presented as the mean ± SD, *n* = 3, **P < 0.01, by the paired *t*-test). **H** Immunofluorescence shows mean fluorescence intensity of 2B4 in uninfected and infected dNK cells (data are presented as the mean ± SD, **P < 0.01, by the paired *t*-test). **I** CD107a expression in uninfected and infected dNK cells with or without anti-2B4 antibody (data are presented as the mean ± SD, *P < 0.05, by the paired *t*-test). **J** LDH release in uninfected and infected dNK cells with or without anti-2B4 antibody

To further investigate the functional correlation between 2B4 expression and the cytokines in human dNK cells found in vivo, PBMCs were isolated from tissues from abortions of healthy early pregnancies and stimulated with *T. gondii* for 20 h. Using flow cytometry, we found that 2B4 expression on the surface of human dNK cells was downregulated during *T. gondii* infection relative to uninfected human dNK cells (Fig. 4a, b). Further, dNK cells were purified by magnetic bead separation; the purification was over 85% (Fig. 4c). There were no differences in the number of dead cells in the uninfected group, infected group, and infected group anti-2B4 antibody-treated infected group (Fig. 4d). In addition, mRNA was isolated to detect *h2B4-A* and *h2B4-B* expression. During *T. gondii* infection, *h2B4-A* expression decreased significantly (Fig. 4e), while protein expression of 2B4 and p-2B4 also decreased after *T. gondii* infection (Fig. 4f). CD48 expression decreased after *T. gondii* infection (Fig. 4g). In addition, in purified human dNK cells stained with DAPI, and APC-conjugated 2B4, immunofluorescence data showed that the mean fluorescence intensity of 2B4 in infected human dNK cells was weaker than that in uninfected human dNK cells (Fig. 4h). The anti-2B4 antibody inhibited CD107a expression during *T. gondii* infection (Fig. 4i). Further, the LDH release assay also showed that cytotoxicity was increased with *T. gondii* infection, while it was significantly downregulated in the presence of the anti-2B4 antibody (Fig. 4j).

### Activation of 2B4 decreases TNF-α and IFN-γ production in human dNK cells after *T. gondii* infection

**Fig. 5:**
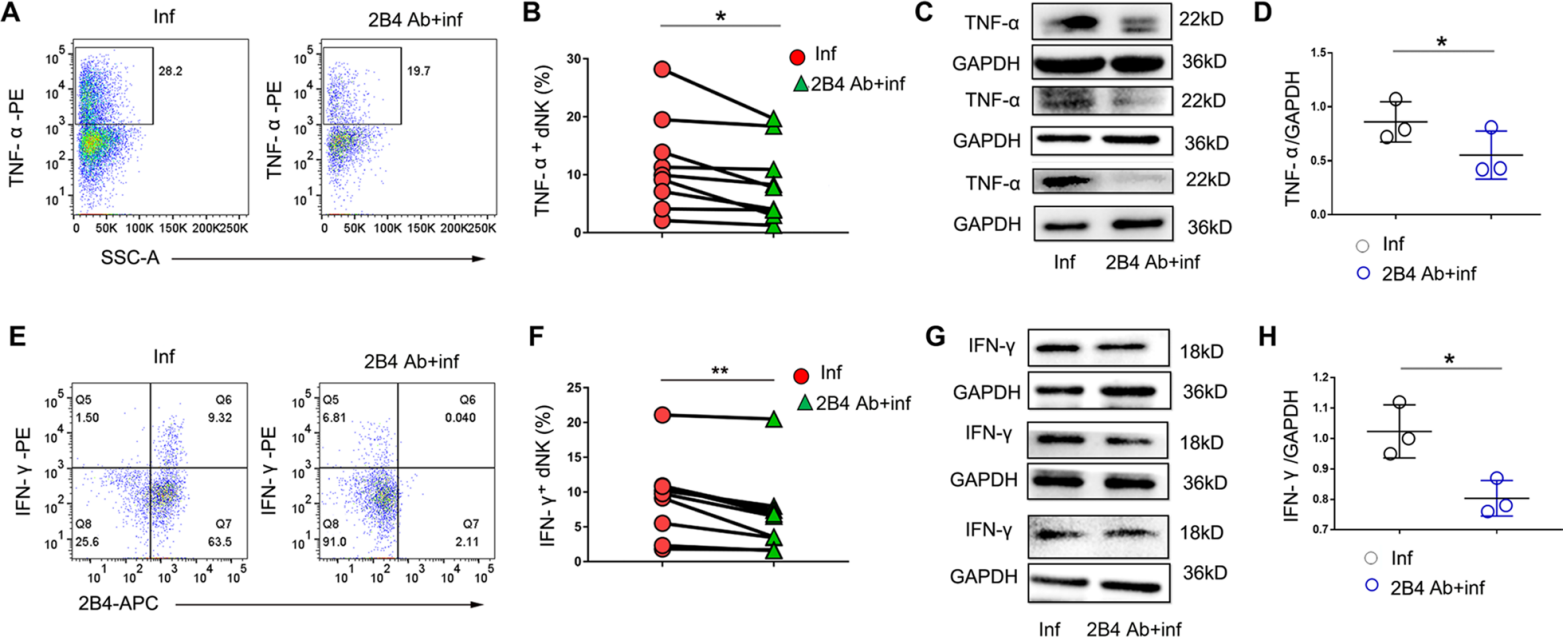
Decreased TNF-α and IFN-γ expression in human dNK cells treated with anti-2B4 antibody following *T. gondii* infection. **A**, **B** The percentage of TNF-α^+^ dNK cells assessed by flow cytometry in *T. gondii*-infected dNK cells with or without anti-2B4 antibody (data are presented as the mean ± SD, *n* = 9, *P < 0.05, by the paired *t*-test). **C**, **D** Western blot of TNF-α expression in *T. gondii*-infected dNK cells with or without anti-2B4 antibody (data are presented as the mean ± SD, *n* = 3, *P < 0.05, by the paired *t*-test). **E**, **F** The percentage of IFN-γ^+^ dNK cells was determined by flow cytometry in *T. gondii*-infected dNK cells with or without anti-2B4 antibody (data are presented as the mean ± SD, *n* = 9, **P < 0.01, by the paired *t*-test). **G**, **H** IFN-γ expression in *T. gondii*-infected dNK cells with or without anti-2B4 antibody pp35 assessed by Western blot (data are presented as the mean ± SD, *n* = 3, *P < 0.05, by the paired *t*-test)

To analyze the relationship between 2B4 and TNF-α or IFN-γ in vitro, we used the anti-2B4 antibody to activate 2B4. The results showed that the addition of the anti-2B4 antibody during *T. gondii* infection activated 2B4 and inhibited TNF-α expression, as shown by flow cytometry (Fig. 5a, b) and Western blotting (Fig. 5c, d). In the presence of the anti-2B4 antibody, the proportion of IFN-γ^+^ dNK cells decreased with *T. gondii* infection compared with that in infected dNK cells without addition of the anti-2B4 antibody (Fig. 5e, f). Western blotting showed that IFN-γ expression in these dNK cells was downregulated upon 2B4 cross-linking after *T. gondii* infection (Fig. 5g, h). Furthermore, 2B4 cross-linking with anti-2B4 antibody did not alter the expression level of KIR2DL4 and CD16 on dNK cells after *T. gondii* infection (Additional file 1: Fig. S3).

### The SHP-2/p-P38 pathway participates in 2B4-induced dysfunction of dNK cells after *T. gondii* infection

**Fig. 6:**
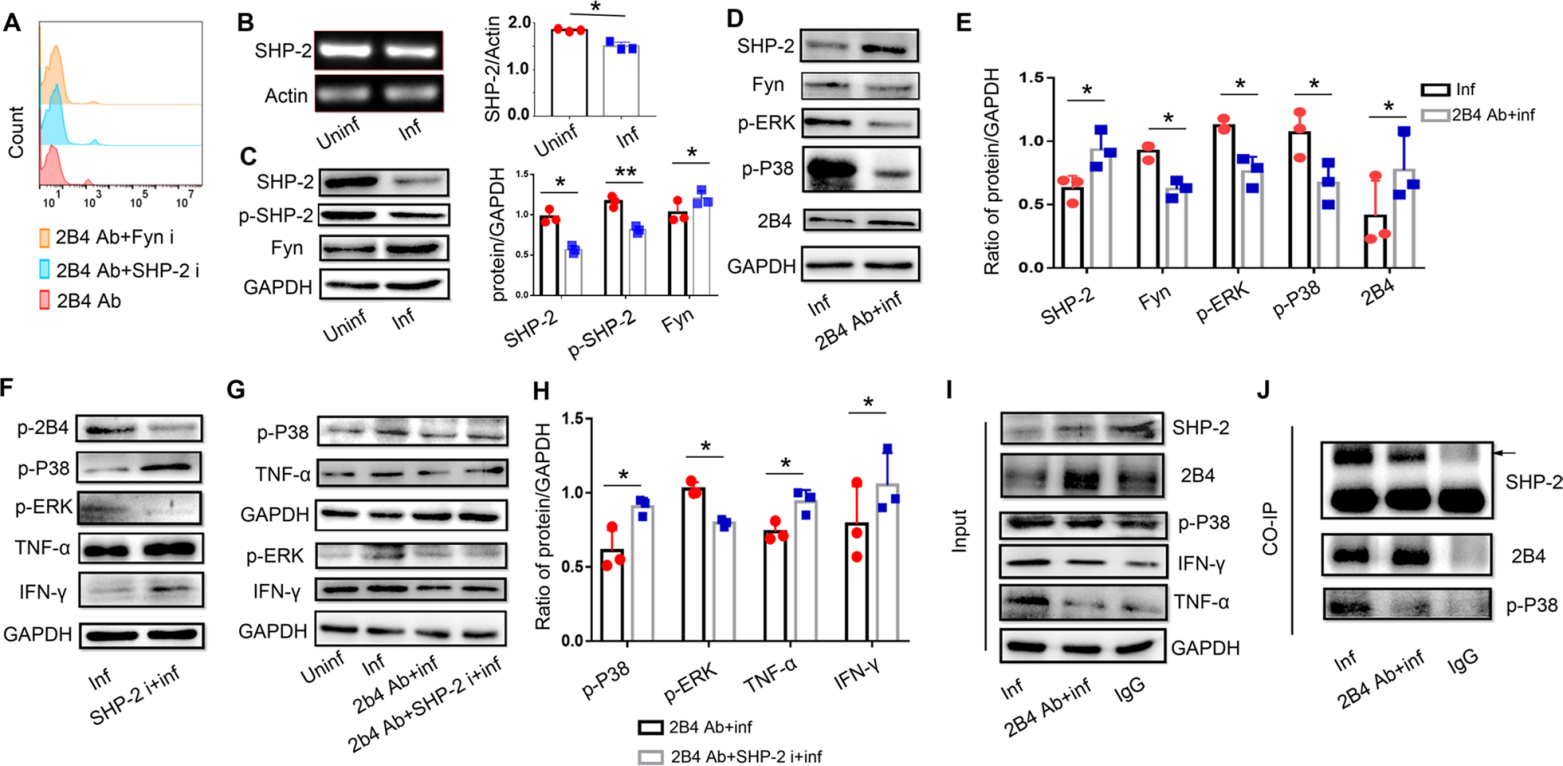
In human dNK cells, the 2B4/SHP-2/p-P38 pathway is involved in 2B4-induced downregulation of TNF-α and IFN-γ after *T. gondii* infection. **A** Some inhibitors may induce cell death, so 7-AAD was used to exclude the effect of SHP-2 inhibitor and Fyn inhibitor on dNK cells. **B** SHP-2 expression in uninfected and infected dNK cells was assessed by PCR (data are presented as the mean ± SD, *n* = 3, *P < 0.05, by the paired *t*-test). **C** SHP-2, p-SHP-2, and Fyn expression in uninfected and infected dNK cells as assessed by Western blot (data are presented as the mean ± SD, *n* = 3, *P < 0.05, **P < 0.01, by the paired *t*-test). **D** Expression of SHP-2, Fyn, p-ERK, p-P38, and 2B4 in infected dNK cells with or without anti-2B4 antibody as assessed by Western blot. **E** Expression of p-P38, p-ERK, TNF-α, IFN-γ, and 2B4 in the infected group with or without anti-2B4 antibody (data are presented as the mean ± SD, *n* = 3, *P < 0.05, by the paired *t*-test). **F** Levels of p-2B4, p-P38, p-ERK, TNF-α, and IFN-γ in the infected group with or without SHP-2 inhibitor treatment. **G** Expression of SHP-2, p-P38, p-ERK, TNF-α, and IFN-γ in uninfected, infected, infected and treated with a 2B4 antibody groups, and infected with 2B4 antibody and SHP-2 inhibitor groups. **H** Analysis for Fig. 6g (data are presented as the mean ± SD, *n* = 3, *P < 0.05, by the paired *t*-test). **I** Input for SHP-2, 2B4, p-P38, IFN-γ, and TNF-α when we used SHP-2 antibody to pull down 2B4 and p-P38. **j** 2B4 and p-P38 were synchronously immunoprecipitated by SHP-2 antibody

Heun et al. reported that SHP-2 activity inversely correlated with the adhesive phenotype of endothelial cells exposed to IL-1β and sepsis serum via p38 mitogen-activated protein kinase (MAPK) [27]. To investigate the role of the SHP-2/p-P38 pathway in 2B4-induced dysfunction of dNK cells after *T. gondii* infection, the anti-2B4 antibody-treated infected group and the anti-2B4 antibody- and SHP-2 inhibitor-treated infected group were constructed. The SHP-2 inhibitor used in this study had no effect on the ratio of dead cells (Fig. 6a). Compared with uninfected dNK cells, we observed a significant reduction in mRNA expression of SHP-2 in infected dNK cells (Fig. 6b). Further, compared with GFP^−^ dNK cells in the infected group, a reduction of SHP-2 was found in GFP^+^ dNK cells (Figure S2c). Compared with uninfected dNK cells, SHP-2 and p-SHP-2 protein expression decreased while Fyn expression increased during *T. gondii* infection (Fig. 6c). Further, compared with GFP^−^ dNK cells in the infected group, upregulation of Fyn was found in GFP^+^ dNK cells (Figure S2C). During *T. gondii* infection, we found that 2B4 activation upregulated 2B4 and SHP-2 expression while downregulating Fyn, p-ERK, and p-P38 expression (Fig. 6d, e). To reveal the involvement of the SHP-2 pathway in 2B4-induced downregulation of TNF-α and IFN-γ expression, we used an SHP-2 inhibitor. The data showed that p-2B4 and p-ERK expression decreased while p-P38, TNF-α, and IFN-γ increased with SHP-2 inhibitor following *T. gondii* infection (Fig. 6f). To further investigate the hypothesis that SHP-2 is involved in downstream 2B4 signaling, we used the SHP-2 inhibitor in the presence of 2B4 following *T. gondii* infection. We found that the SHP-2 inhibitor upregulated the p-P38, TNF-α, and IFN-γ expression while downregulating the p-ERK expression in the presence of the anti-2B4 antibody after *T. gondii* infection (Fig. 6g, h). Co-IP data showed synchronous binding of 2B4 and p-P38 by SHP-2 (Fig. 6i, j).

### The Fyn/p-ERK pathway participates in 2B4-induced dysfunction of dNK cells after *T. gondii* infection

**Fig. 7:**
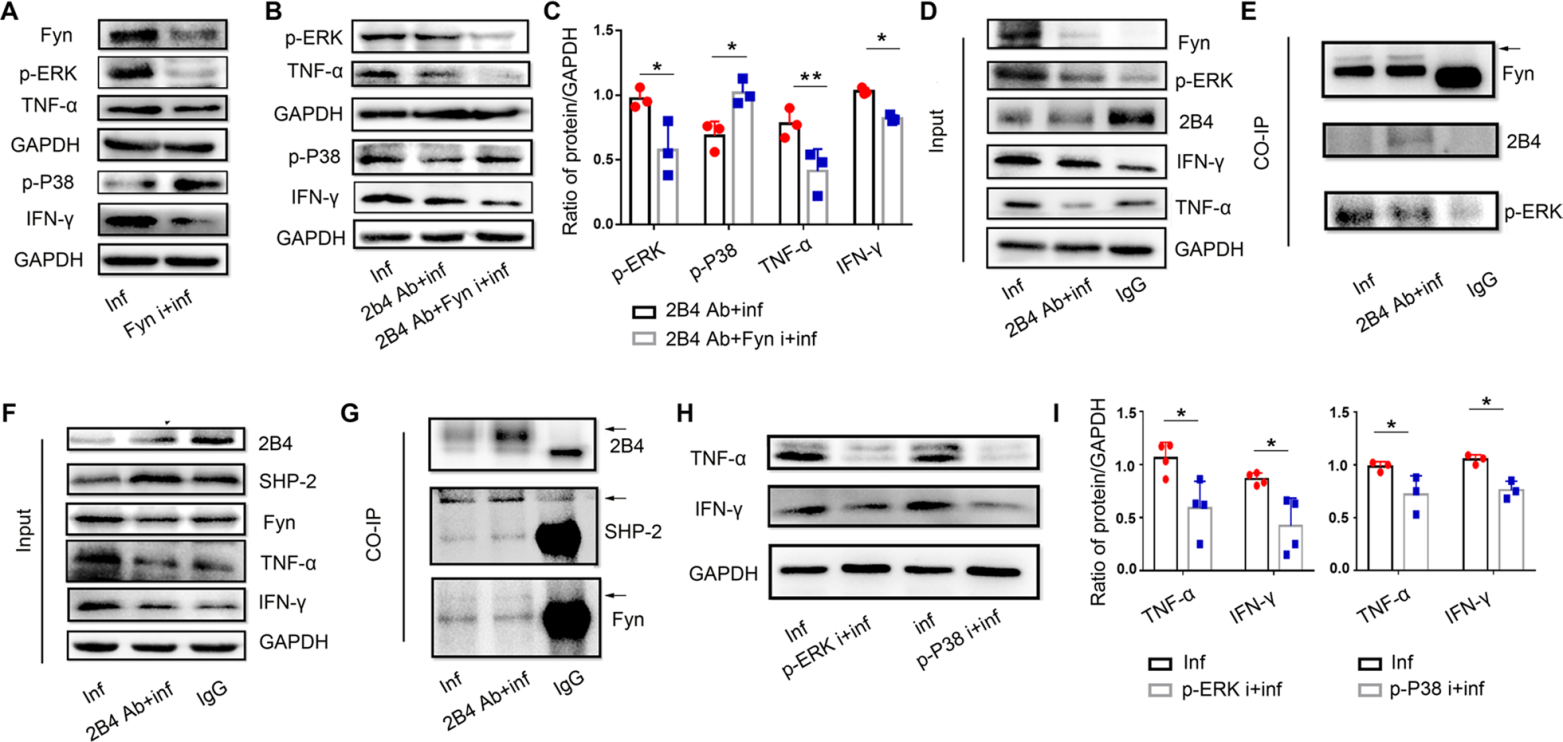
In human dNK cells, the 2B4/Fyn/p-ERK pathway is involved in 2B4-induced downregulation of TNF-α and IFN-γ after *T. gondii* infection. **A** Fyn, p-ERK, TNF-α, p-P38, and IFN-γ expression in the infected group with or without Fyn inhibitor treatment. **B** Expression of p-ERK, TNF-α, p-P38, and IFN-γ in the infected group, infected group treated with the 2B4 antibody, and the infected group treated with both anti-2B4 antibody and the Fyn inhibitor. **C** Analysis for Fig. 7b (data are presented as the mean ± SD, *n* = 3, **P < 0.01, *P < 0.05, by the paired *t*-test). **D** Input for Fyn, p-ERK, 2B4, IFN-γ, and TNF-α when we used Fyn to bind 2B4 and p-ERK. **E** 2B4 and p-ERK were synchronously immunoprecipitated by Fyn antibody. **F** Input for 2B4, SHP-2, Fyn, TNF-α, and IFN-γ when we used 2B4 to bind to SHP-2 or Fyn. **G** SHP-2 and Fyn were synchronously immunoprecipitated by 2B4 antibody. **H** Expression of TNF-α and IFN-γ in infected dNK cells with or without p-ERK inhibitor or p-P38 inhibitor, respectively. **I** Analysis of Fig. 7h (data are presented as the mean ± SD, *n* = 3, *P < 0.05, by the paired *t*-test)

We used a Fyn inhibitor to analyze the role of Fyn in the 2B4-induced cytotoxicity of dNK cells during *T. gondii* infection. Compared with infected dNK cells, Fyn, p-ERK, TNF-α, and IFN-γ expression downregulated while p-P38 expression increased after *T. gondii* infection in the presence of the Fyn inhibitor (Fig. 7a). Upon further probing to understand the involvement of the 2B4/Fyn pathway in 2B4-induced downregulation of TNF-α and IFN-γ expression, we found that the Fyn inhibitor downregulates p-ERK, TNF-α, and IFN-γ expression while upregulating p-P38 expression in the presence of the anti-2B4 antibody following *T. gondii* infection (Fig. 7b, c). Co-IP data showed that the Fyn antibody synchronously binds 2B4 and p-ERK (Fig. 7d, e).

### 2B4 affects downstream signaling by binding to Fyn and SHP-2 directly

To further study the relationship between 2B4 and its downstream signaling, we used the 2B4 IP antibody. Inputs for 2B4, SHP-2, Fyn, TNF-α, and IFN-γ were detected, and similar results were found as in Fig. 7d. Co-IP data showed that the 2B4 antibody binds to 2B4 molecular and also binds to SHP-2 or Fyn in dNK cells directly (Fig. 7f, g).

### p-ERK and p-P38 affect TNF-α and IFN-γ expression in dNK cells following *T. gondii* infection

To explore the involvement of the 2B4/SHP-2/p-P38 and 2B4/Fyn/p-ERK pathways in TNF-α and IFN-γ expression in dNK cells after *T. gondii* infection, we used p-ERK and p-P38 inhibitors, respectively. We found that TNF-α and IFN-γ expression decreased in the presence of the p-ERK inhibitor or in the presence of the p-P38 inhibitor during *T. gondii* infection (Fig. 7h, i).

### Proposed mechanistic model

**Fig. 8:**
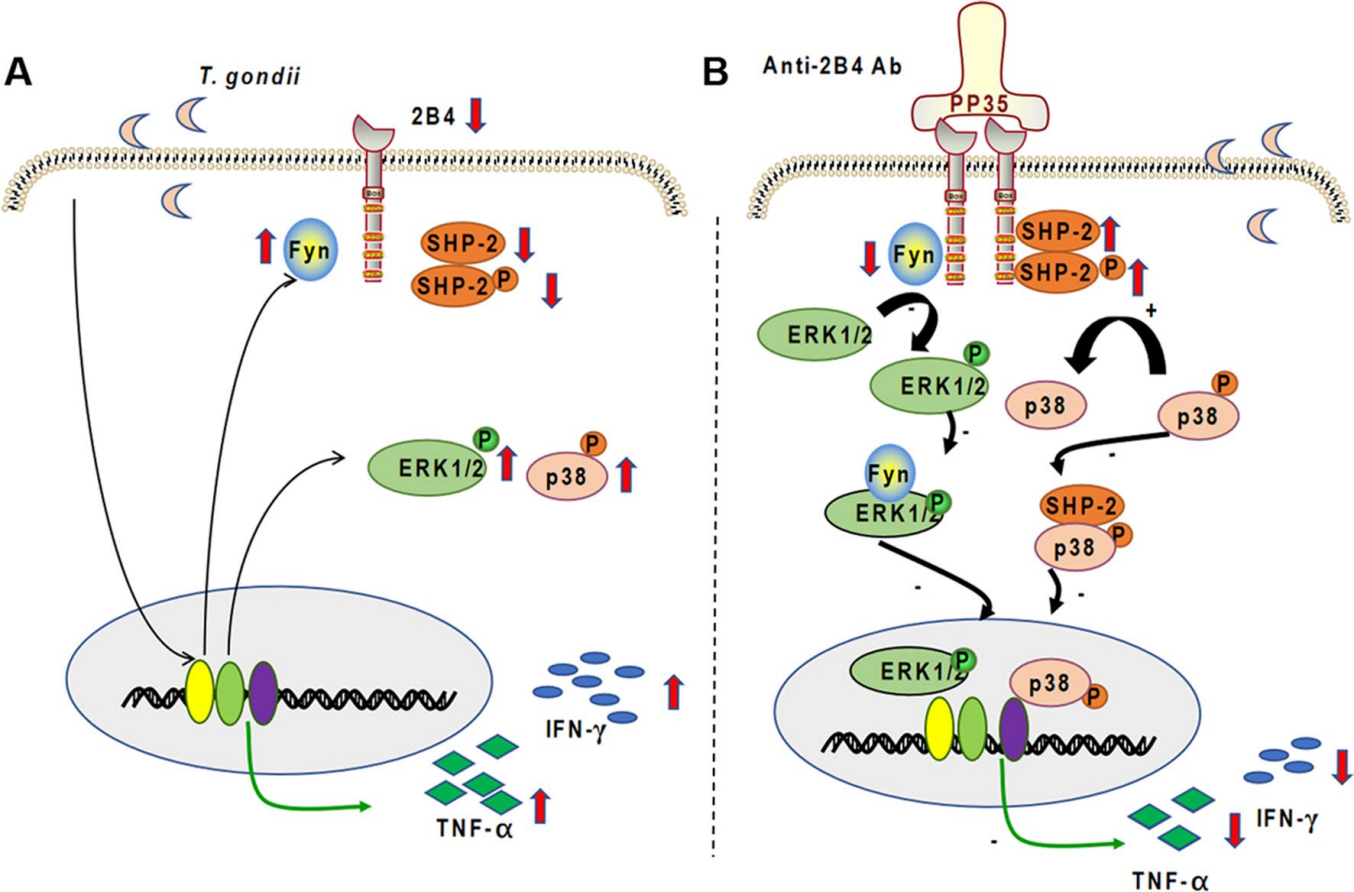
Working model for the roles of 2B4 in dNK cells following *T. gondii* infection. **A** In summary, the expressions of 2B4, SHP-2, p-SHP-2, Fyn, p-ERK, p-P38, TNF-α, and IFN-γ were found in *T. gondii*-infected dNK cells relative to uninfected dNK cells. **B** After 2B4 cross-linking, activation of 2B4 can downregulate TNF-α and IFN-γ expression by activating the 2B4/SHP-2/p-P38 pathway and inhibiting the 2B4/Fyn/p-ERK pathway following *T. gondii* infection

*Toxoplasma gondii* infection downregulates 2B4, SHP-2, and p-SHP-2 levels and upregulates Fyn, p-ERK, p-P38, IFN-γ, and TNF-α expression in dNK cells (Fig. 8a). As a surface receptor, 2B4 also binds SHP-2 and Fyn, which triggers activation of different signaling pathways to regulate expression of TNF-α and IFN-γ in dNK cells. In addition, SHP-2 binds 2B4 upstream and binds to p-P38 downstream with 2B4 cross-linking, thereby transmitting an inhibitory signal to TNF-α and IFN-γ during *T. gondii* infection. Fyn also binds 2B4 upstream and p-ERK downstream with 2B4 cross-linking, representing TNF-α and IFN-γ expression in dNK cells after *T. gondii* infection (Fig. 8b).

## Discussion

Some studies have suggested the involvement of 2B4 in anti-infection, maintenance of persistent infection, and tumor escape [24, 28, 29]. Moreover, one study showed that changes in 2B4 expression in the peripheral blood was related to sporadic miscarriage [30]. However, there have been no studies detailing the role of 2B4 in pregnancy failure after *T. gondii* infection, and the mechanism(s) behind 2B4 in adverse pregnancy following *T. gondii* infection, until now, have remained unclear. In this study, we found that 2B4 expression in mouse decidual immune cells, and especially on dNK cells, was significantly downregulated after *T. gondii* infection. We also provide evidence that infected pregnant 2B4^−/−^ mice exhibited worse pregnancy outcomes than their WT counterparts. These data suggest a role for the inhibitory receptor 2B4 as another important immune molecule in adverse pregnancy outcomes, also making it an important mediator for other forms of pregnancy failure, in addition to the *T. gondii* model. Consequently, further research is required to gain detailed insights into the involvement of 2B4 in *T. gondii*-induced pregnancy failure and abnormal pregnancy outcomes. The findings in this work lay a good foundation for the discovery of additional important functional molecules in pregnancy failure. Also, in this work, infected human dNK cell models were established and showed that expression of 2B4, p-2B4, and its ligand CD48 in human dNK cells decreased in infected cells relative to uninfected cells. These results further suggest that the decreased expression of 2B4 itself or stimulation by CD48 may be responsible for 2B4 pathway activation during *T. gondii* infection. However, elucidation of the detailed mechanism requires further exploration.

A previous study suggested that engagement of 2B4 in different cell types and the stage of differentiation may lead to different outcomes. In addition, 2B4 has been reported as an inhibitory receptor during the early stages of NK cell differentiation [31], possibly ensuring self-tolerance of developing NK cells. One study indicated that NK cytotoxicity of dNK cells can be inhibited by 2B4 [32].

IFN-γ is a key Th1-type cytokine that can be secreted by dNK cells at the maternal–fetal interface, and plays an important role in controlling excessive trophoblast invasion in early implantation [33]. Higher IFN-γ can induce apoptosis of trophoblast cells and lead to fetal damage during *T. gondii* infection [34]. The increases in TNF-α in pregnant mice resulted in worse pregnancy outcomes by affecting vertical transmission of *T. gondii* [35]. IFN-γ-activated macrophages inhibit the replication of intracellular parasites and disrupt the integrity of vacuolar pathogens [36]. The role of TNF-α in *T. gondii* infection is still controversial; however, it has been reported that TNF-α can facilitate the intracerebral spread of *T. gondii* in mice [37]. In our previous study, results showed that *T. gondii* infection could induce increased TNF-α and IFN-γ production in dNK cells [17]. The question whether 2B4 can affect TNF-α and IFN-γ expression during *T. gondii* infection warrants further exploration. Here, we observed a significant increase in TNF-α and IFN-γ production in dNK cells in infected 2B4^−/−^ mice compared with that in infected WT mice, with a concurrent increase in cytotoxicity. These results suggest that 2B4 knockout is related to dNK cell activation during *T. gondii* infection, which may manifest as upregulated TNF-α and IFN-γ production. Next, we used the anti-2B4 antibody to further investigate the role of 2B4 in TNF-α and IFN-γ expression in human dNK cells. Our data show that cross-linking of 2B4 downregulates TNF-α and IFN-γ expression in dNK cells, leading to inactivation of dNK cell cytotoxicity during *T. gondii* infection. These results confirm that downregulation of 2B4 expression by *T. gondii* infection may induce pregnancy failure via enhanced dNK cell cytotoxicity. However, the detailed mechanism(s) in the downstream 2B4 pathway require further investigation.

Apart from the level of 2B4, adaptor molecule and competitive binding experiments can also determine whether CD244 propagates an activating or inhibitory signal [38]. It was shown that 2B4 recruits SHP-2 and phosphatase SHP-2 (p-SHP-2), leading to the propagation of inhibitory signals [39]. SHP-2 has also been shown to inhibit lipopolysaccharide-mediated TNF-α and IFN-γ production in murine macrophages [40]. In this study, SHP-2 and p-SHP-2 expression in dNK cells decreased after *T. gondii* infection, while TNF-α and IFN-γ expression increased in the presence of an anti-2B4 antibody with an SHP-2 inhibitor after *T. gondii* infection compared with the same conditions without an SHP-2 inhibitor. These data suggest that *T. gondii* infection may block the 2B4/SHP-2 signaling pathway to induce dysfunction of dNK cells after *T. gondii* infection. The mechanism(s) behind 2B4/SHP-2-mediated inhibition of TNF-α and IFN-γ require further study.

SHP-2-deficient NK cells were significantly downregulated, while phosphorylation of ERK was abrogated [41]. It was shown that the polysaccharide isolated from *Ferula gummosa* (FGP) could upregulate the expression of TNF-α and IFN-γ in NK-92 cells via activation of p-ERK [42]. These results suggest that p-ERK may be involved in 2B4/SHP-2 pathway-induced TNF-α and IFN-γ expression. Surprisingly, when p-ERK expression decreased with 2B4 cross-linking following *T. gondii* infection, the SHP-2 inhibitor also decreased p-ERK expression, which was inconsistent with the observed expression of TNF-α and IFN-γ in dNK cells. These results suggest that 2B4 may regulate TNF-α and IFN-γ expression in dNK cells via the 2B4/SHP-2 pathway but not through ERK phosphorylation after *T. gondii* infection. Further, p38 MAPK recruits SHP-1 replication to facilitate SHP-1-induced dephosphorylation of p38 MAPK, and thereby attenuates p38 MAPK activation and hepatitis B virus (HBV) replication [43]. In this study, we show that SHP-2 inhibition with or without 2B4 cross-linking can significantly increase the level of p-P38 and simultaneously upregulate TNF-α and IFN-γ expression after *T. gondii* infection. These results indicate that downregulation of 2B4 after *T. gondii* infection may affect p-P38 via the 2B4/SHP-2 pathway, leading to high TNF-α and IFN-γ expression during *T. gondii* infection. In addition, inhibition of p-P38 can lead to downregulation of TNF-α and IFN-γ expression in dNK cells, which suggests that TNF-α and IFN-γ expression in dNK cells may be regulated by the 2B4/SHP-2/p-P38 signaling pathways following *T. gondii* infection. Co-IP assays indicated that SHP-2 binds to 2B4 as well as p-P38 directly during *T. gondii* infection. Taken together, our data explicitly show that activation of 2B4 can reduce TNF-α and IFN-γ expression via the 2B4/SHP-2/p-P38 pathways, ultimately resulting in dNK cell dysfunction.

Fyn is a member of the Src family of non-receptor protein tyrosine kinases, which is highly expressed in NK cells and plays an important role in lymphocyte activation, cytotoxicity, and inflammatory cytokine production [44]. By analyzing mice deficient in Fyn, we found that Fyn plays an important role in mediating the 2B4 function in dNK cells [45]. In addition, Fyn regulates TNF-α release from bone marrow-derived mast cells via modulation of ERK phosphorylation [46]. Whether 2B4 affects Fyn, then disturbs p-ERK, triggering TNF-α and IFN-γ expression during *T. gondii* infection, needs to be further investigated. Moreover, we show evidence that Fyn inhibition impairs p-ERK, TNF-α, and IFN-γ expression during *T. gondii* infection with 2B4 cross-linking. These results demonstrate that 2B4 cross-linking may regulate the expression of TNF-α and IFN-γ through the 2B4/Fyn/p-ERK pathway after *T. gondii* infection. Co-IP assays showed that the Fyn antibody could bind to itself and also interact with 2B4 and p-ERK directly. In addition, the binding to 2B4 increased while binding to p-ERK deceased. Taken together, these results provide evidence that the 2B4/Fyn/p-ERK signaling pathway is a potent activating pathway of natural cytotoxicity in dNK cells.

## Conclusions

In summary, downregulation of 2B4 caused by *T. gondii* infection led to dNK cell dysfunction via the 2B4/SHP-2/p-P38 and 2B4/Fyn/p-ERK pathways, which may be a vital mechanism involved in adverse pregnancy outcomes during *T. gondii* infection. Thus, 2B4, a novel immune inhibitory receptor, may be involved in pathogen-induced pregnancy failure as well as in other forms of pregnancy loss. Therefore, 2B4 is a promising novel therapeutic target for pregnancy diseases.

## Supplementary Information

**Additional file 1: Figure S1.** Generation of the mouse model. (a) WT and 2B4^−/−^ mice primers for identification were constructed. (b) The WT mice were identified, and the specific gene product length is 280 bp. (c) The 2B4^−/−^ mice were identified, and the specific gene product length is 430 bp.

**Additional file 2: Figure S2.** The GFP-*T. gondii *RH strain was received as a gift from Anhui Medical University, and dNK cells were collected, purified, and infected with the GFP-*T. gondii *RH strain. (a) The *T. gondii* invasion process was imaged using the Live Cell Workstation. (a1) GFP^+^ *T. gondii,* indicated by red arrows, is located outside the dNK cells. (a2) GFP^+^ *T. gondii* is in close contact with the dNK cell membrane. (a3) GFP^+^ *T. gondii *continues to penetrate the dNK cell membrane. (a4) GFP^+^*T. gondii *passes through the membrane to invade dNK cells. Green represents *T. gondii*, red represents the dNK cell membrane, and the arrows represent the process of *T. gondii* invasion. (b) dNK cells were divided into two groups; uninfected group and infected group. The infected cells containing *Toxoplasma* were separated by flow cytometry. (c) Expression of 2B4, SHP-2, or Fyn in the uninfected group. GFP^-^ dNK cells and GFP^+^ dNK cells in the infected group were detected by PCR.

**Additional file 3: Figure S3.** Fyn has been reported to affect other activating receptors such as LIR2DL4 and CD16. (a, b) KIR2DL4 expression in infected dNK cells in the absence and presence of the anti-2B4 antibody pp35 was detected by flow cytometry (data are presented as the mean ± SD, *n* = 3, *P* > 0.05, by the paired *t*-test). (c, d) CD16 expression in *T. gondii*-infected dNK cells with or without the anti-2B4 antibody as detected by flow cytometry (data are presented as the mean ± SD, *n* = 3, *P* > 0.05, by the paired *t*-test).

## Data Availability

The data that support the findings of this study are available from the corresponding author upon reasonable request.

## Abbreviations

dNK: Decidual natural killer
*T. gondii*: *Toxoplasma gondii*
TNF-α: Tumor necrosis factor alpha
IFN-γ: Interferon gamma
HFF: Human foreskin fibroblast
WT: Wild-type
2B4^−/−^: 2B4 knockout
PBS: Phosphate-buffered saline
