# Supplementary figures and images for: A novel 2B4 receptor leads to worse pregnancy outcomes by facilitating TNF-α and IFN-γ production in dNK cells during Toxoplasma gondii infection
Source: Parasit Vectors. 2022 Sep 24;15:337. doi: 10.1186/s13071-022-05455-9 (PMC9509566; doi:10.1186/s13071-022-05455-9)

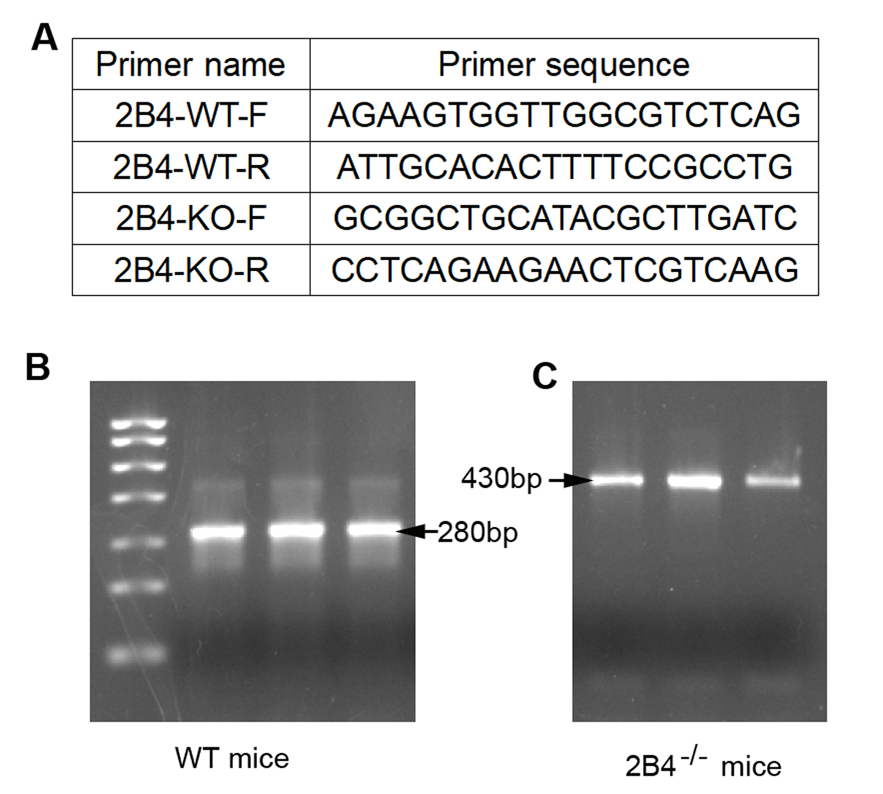

Supplement: Supplementary file 1 — Additional file 1: Figure S1. Generation of the mouse model. (a) WT and 2B4−/− mice primers for identification were constructed. (b) The WT mice were identified, and the specific gene product length is 280 bp. (c) The 2B4−/− mice were identified, and the specific gene product length is 430 bp. [file 13071_2022_5455_MOESM1_ESM.tif]

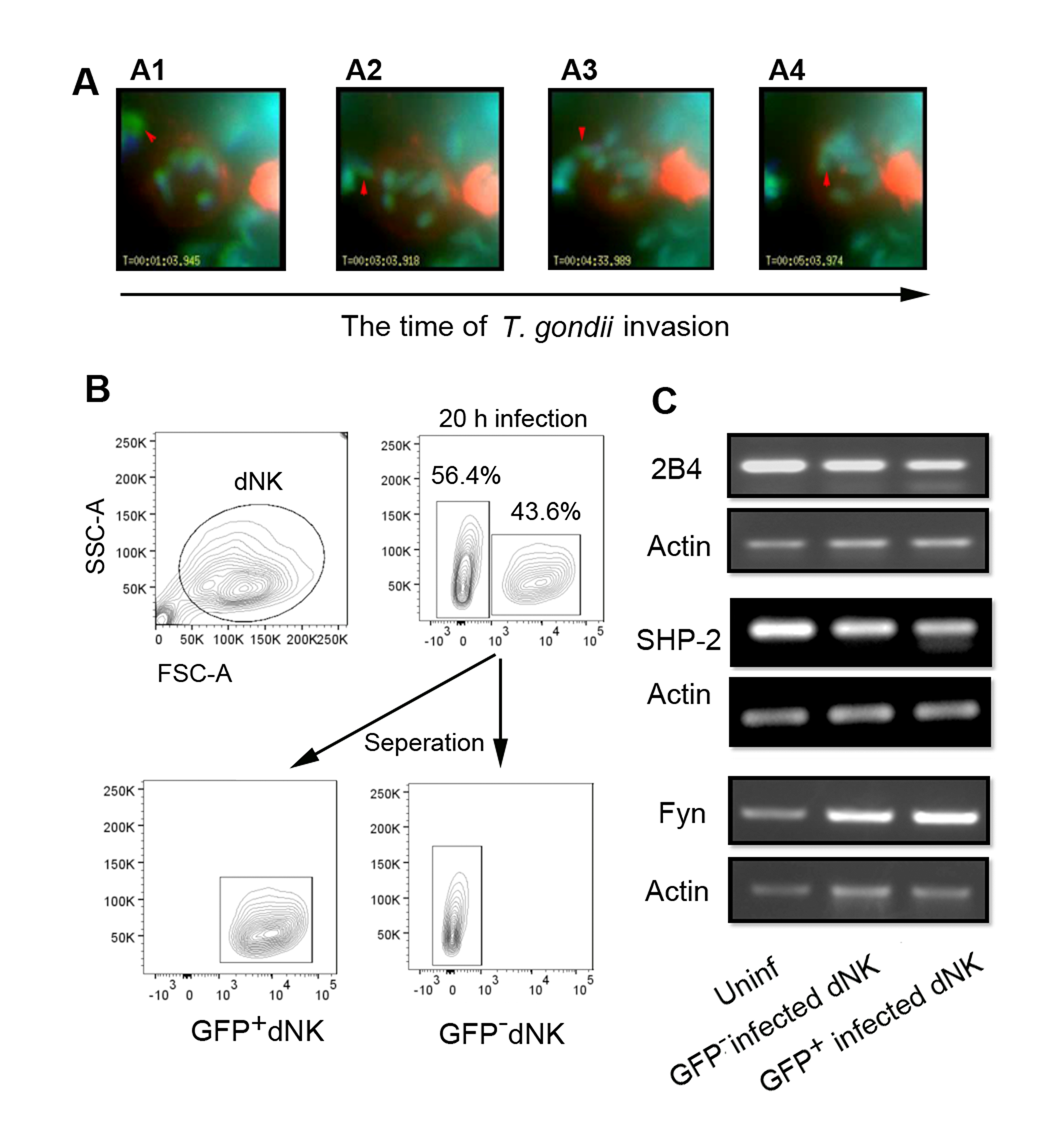

Supplement: Supplementary file 2 — Additional file 2: Figure S2. The GFP-T. gondii RH strain was received as a gift from Anhui Medical University, and dNK cells were collected, purified, and infected with the GFP-T. gondii RH strain. (a) The T. gondii invasion process was imaged using the Live Cell Workstation. (a1) GFP+ T. gondii, indicated by red arrows, is located outside the dNK cells. (a2) GFP+ T. gondii is in close contact with the dNK cell membrane. (a3) GFP+ T. gondii continues to penetrate the dNK cell membrane. (a4) GFP+T. gondii passes through the membrane to invade dNK cells. Green represents T. gondii, red represents the dNK cell membrane, and the arrows represent the process of T. gondii invasion. (b) dNK cells were divided into two groups; uninfected group and infected group. The infected cells containing Toxoplasma were separated by flow cytometry. (c) Expression of 2B4, SHP-2, or Fyn in the uninfected group. GFP- dNK cells and GFP+ dNK cells in the infected group were detected by PCR. [file 13071_2022_5455_MOESM2_ESM.tif]

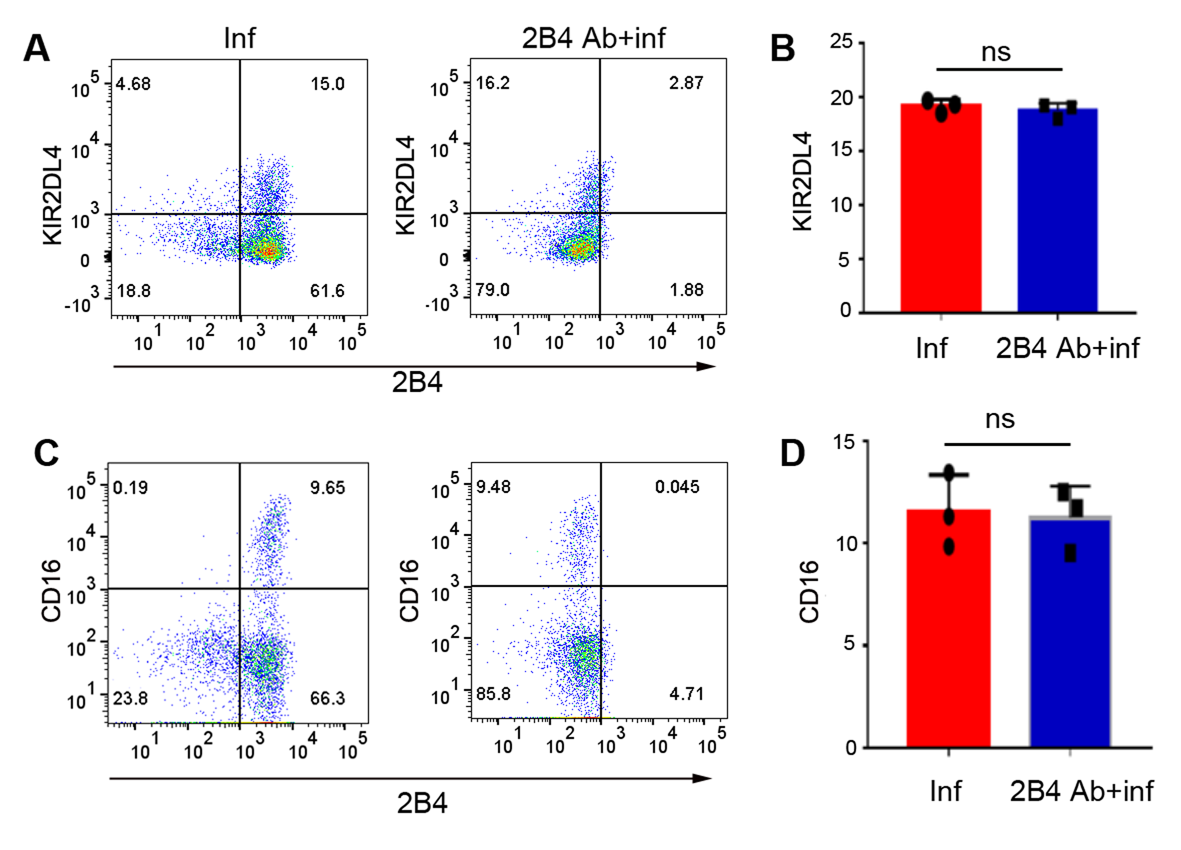

Supplement: Supplementary file 3 — Additional file 3: Figure S3. Fyn has been reported to affect other activating receptors such as LIR2DL4 and CD16. (a, b) KIR2DL4 expression in infected dNK cells in the absence and presence of the anti-2B4 antibody pp35 was detected by flow cytometry (data are presented as the mean ± SD, n = 3, P > 0.05, by the paired t-test). (c, d) CD16 expression in T. gondii-infected dNK cells with or without the anti-2B4 antibody as detected by flow cytometry (data are presented as the mean ± SD, n = 3, P > 0.05, by the paired t-test). [file 13071_2022_5455_MOESM3_ESM.tif]
